# Prognostic Factors in Endodontic Surgery Using an Endoscope: A 1 Year Retrospective Cohort Study

**DOI:** 10.3390/ma15093353

**Published:** 2022-05-07

**Authors:** Shintaro Sukegawa, Rieko Shimizu, Yuka Sukegawa, Kazuaki Hasegawa, Sawako Ono, Ai Fujimura, Izumi Yamamoto, Keisuke Nakano, Kiyofumi Takabatake, Hotaka Kawai, Hitoshi Nagatsuka, Yoshihiko Furuki

**Affiliations:** 1 Department of Oral and Maxillofacial Surgery, Kagawa Prefectural Central Hospital, 1-2-1 Asahi-machi, Takamatsu 760-8557, Japan; de421021@s.okayama-u.ac.jp (R.S.); yuka611225@gmail.com (Y.S.); de421040@s.okayama-u.ac.jp (K.H.); sugar.x.48@gmail.com (A.F.); iyamamoto8408@gmail.com (I.Y.); furukiy@ma.pikara.ne.jp (Y.F.); 2 Department of Oral Pathology and Medicine, Okayama University Graduate School of Medicine, Dentistry and Pharmaceutical Sciences, Okayama 700-8525, Japan; keisuke1@okayama-u.ac.jp (K.N.); gmd422094@s.okayama-u.ac.jp (K.T.); de18018@s.okayama-u.ac.jp (H.K.); jin@okayama-u.ac.jp (H.N.); 3 Department of Pathology, Kagawa Prefectural Central Hospital, Takamatsu 760-8557, Japan; de19008@s.okayama-u.ac.jp

**Keywords:** retrospective cohort study, endoscope, endodontic surgery, prognostic factors

## Abstract

This retrospective study clarified the success rate of endoscopic endodontic surgeries and identified predictors accounting for successful surgeries. In this retrospective study, 242 patients (90 males, 152 females) who underwent endoscopic endodontic surgery at a single general hospital and were diagnosed through follow-up one year later were included. Risk factors were categorized into attributes, general health, anatomy, and surgery. Then, the correlation coefficient was calculated for the success or failure of endodontic surgery for each variable, the odds ratio was calculated for the upper variable, and factors related to the surgical prognosis factor were identified. The success rate of endodontic surgery was 95.3%, showing that it was a highly predictable treatment. The top three correlation coefficients were post, age, and perilesional sclerotic signs. Among them, the presence of posts was the highest, compared with the odds ratio, which was 9.592. This retrospective study revealed the success rate and risk factors accounting for endoscopic endodontic surgeries. Among the selected clinical variables, the presence of posts was the most decisive risk factor determining the success of endodontic surgeries.

## 1. Introduction

Endodontic surgery is selected when conservative treatments are considered infeasible or re-root canal treatment fails [1], making it an important option during modern endodontic treatment [2]. Recently, the high success rate of endodontic surgeries has come from introducing microscopes, advanced technologies, and tools, including ultrasonic instruments, biocompatible root-end filling materials, and miniaturized equipment [3,4,5,6].

Alternatively, microsurgery is a surgical procedure performed on tiny and complex tissue structures. This procedure can be achieved using a dental microscope or endoscope [7]. Studies [8,9] have shown that microsurgery is useful for microscopic endodontic surgeries. However, this procedure has limited mobility and requires advanced mirroring techniques, depending on the surgical site. Additionally, although it is useful for specialists, it lacks generalizability.

In contrast, a typical endoscopic surgery is easy to perform because it involves no limitations in mobility and the endoscope can be inserted in various directions. Especially for oral and maxillofacial surgeons, endoscopic surgery is also used in daily surgery and is easy to introduce. However, it is not a complete treatment method, even during micro-endodontic surgeries using an endoscope, the probability of which is increasing. Additionally, other than surgical factors, other factors accounting for the success of endoscopic surgeries are not yet clear. Therefore, this study clarified the success rate of endoscopic endodontic surgeries and identified predictors of successful surgeries.

## 2. Materials and Methods

### 2.1. Study Design

This retrospective cohort study was conducted between April 2011 and March 2020. The study involved patients who underwent endodontic surgery using an endoscope, extracted through oral and maxillofacial surgery, at the Kagawa Prefectural Central Hospital, Takamatsu, Japan.

### 2.2. Ethics Statement

The institutional review committee of Kagawa Prefectural Central Hospital (approval number 1045, approved on 12 July 2021) approved this study. Furthermore, this study adopted a non-interventional retrospective design, removing the need for individual informed consent, after which all data were analyzed anonymously.

### 2.3. Patients

The following criteria were adopted for the case selection. First, patients whose teeth were treated endodontically and who subsequently experienced cystic lesions of endodontic origin larger than 3 mm were included. Furthermore, those in whom nonsurgical retreatment was considered infeasible or the procedure had failed previously were also included. Additionally, patients whose surgically treated tooth showed satisfactory final restoration with no clinical evidence of coronal leakage, including those experiencing no acute symptom, such as pus leakage at the time of surgery, were included. Moreover, we included patients experiencing no general medical limitations related to oral and maxillofacial surgery based on expected patient conditions. Similarly, patients who gave informed consent for endodontic surgery and met the selection criteria for postoperative follow-up at our institution to evaluate the outcome were included in the study.

The following exclusion criteria were applied: (1) patients whose teeth had lesions associated with root fractures were excluded, including those with cysts contiguous with the inferior alveolar nerve in molars and whose teeth had severe periodontal bone loss with extreme mobility; (2) systemically, pregnant women and patients with previous allergic reactions to antibiotics, including those with osteoporosis or malignancy who were taking antiangiogenic drugs or antiresorptive agents, such as bisphosphonates or antireceptor activators of NF-κB ligand monoclonal antibodies, were excluded; (3) patients with malignant tumors who had received radiation therapy to the head and neck region were also excluded. According to the above criteria, 362 teeth in 242 consecutive patients were included in the study.

### 2.4. Surgical Procedure

All surgical procedures were conducted under local anesthesia (2% lidocaine solution with 12.5 microgram/mL of epinephrine) with intravenous sedation with midazolam. Additionally, a single surgical team comprising one expert oral and maxillofacial surgeon (S.S.) performed the apical surgery.

**Figure 1:**
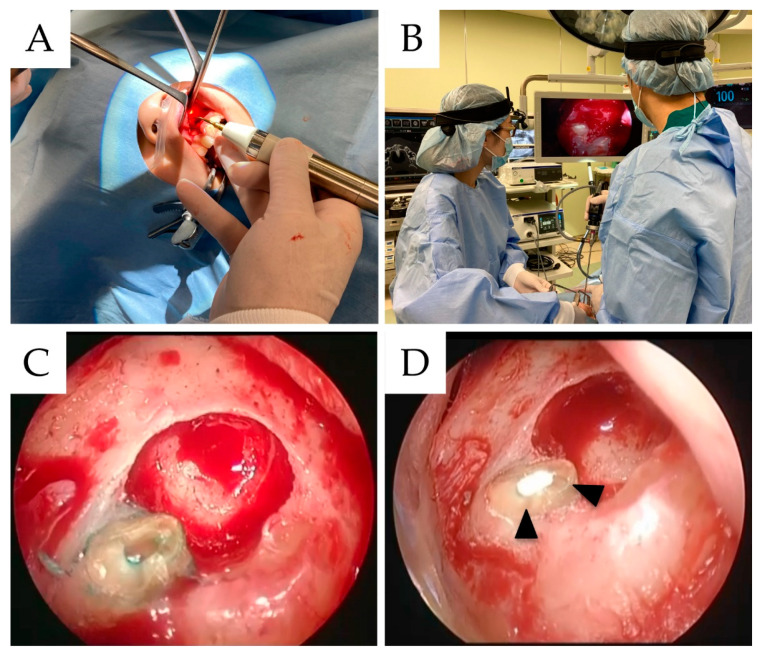
(**A**) The piezosurgery was used for root-end canal cleaning. (**B**) The image shows an endoscopic endodontic surgery. The surgical field of view magnified by the endoscope through the large screen monitor in the operating room is shown. (**C**) Confirmation of presence or absence of microcracks after staining with methylene blue. (**D**) MTA cement was filled in the root-end apex. (arrowhead: MTA cement).

First, a horizontal submarginal incision was made 3–4 mm from the gingival margin. Then, an incision was made perpendicular to each distal end of the causative tooth, after which the full thickness of the mucosal periosteal flap was reflected and retracted. Peri-lesion bone removal provided surgical access to the root through the cortical bone using a round bar for complete visualization of the lesion and root apex. Furthermore, this operation was conducted under constant large quantities of sterile water irrigation. Then, peri-root cystic-like lesions were removed with a sharp bone curette. The cut root-end tip attached with a cystic sac was sent to the Department of Oral Pathology to identify the etiology of apical pathosis. The curetted tissue was later placed in 10% formalin for subsequent pathological diagnosis. After exposing the apex, an ultrasound device (Piezosurgery Touch, Mectron, Italy) was inserted into the handpiece perpendicular to the long axis of the root, and the end of the root was scraped for 3 mm. Additionally, a 4 mm endoscope ((Visera Elite II Video System, Olympus, Tokyo, Japan) and (7230BWA, Hopkins Telescope, Karl Storz, Germany)) was inserted into the apex using the first assistant, after which subsequent procedures were performed from an endoscopic field of view (Figure 1). Depending on the case, a slice cut angle at the endoscope tip of 0, 30, or 70 degrees was selected. The cavity at a depth of 3 mm from the apical resection was also prepared under constant copious irrigation with sterile water using an ultrasonic device retro-tip. This tip had an angled shaft (Retrograde Microsurgery, Mectron, Italy), driven using an ultrasonic device.

Subsequently, the root cavities were dried and careful examination detected root morphological changes, such as edge chipping and the presence of isthumus or accesory canals or any presence of microcracks using methylene blue staining. Then, retrograde cavities of both teeth were filled with MTA (ProRoot MTA, Dentsply Sirona, New York, NY, USA). Afterward, excess filling material was removed if necessary, followed by autologous bone collection from the mandibular ramus, transplantation to the donor site at the lesion excision site, and filling of the excision fossa. Finally, the reflexed tissue was reapproached to its original position, compressed, stabilized, and sutured with absorbent silk 4–0 (Surgisorb 4–0; Nicho Co., Ltd., Tokyo, Japan).

The same procedure and postoperative clinical management were used for all patients. Furthermore, all patients were administered intravenous antibiotics (Cefazolin sodium, 1.0 g) during the surgery. Likewise, patients received antibiotics (Amoxicillin hydrate, 250 mg) every 8 h for 2 days after the operation. They also received nonsteroidal anti-inflammatory analgesics (Celecoxib, 400 mg (for initial pain) or 200 mg every 6 h (for sec episode or later), or Acetaminophen, 500 mg every 6 h)).

### 2.5. Predictor Variables

**Figure 2:**
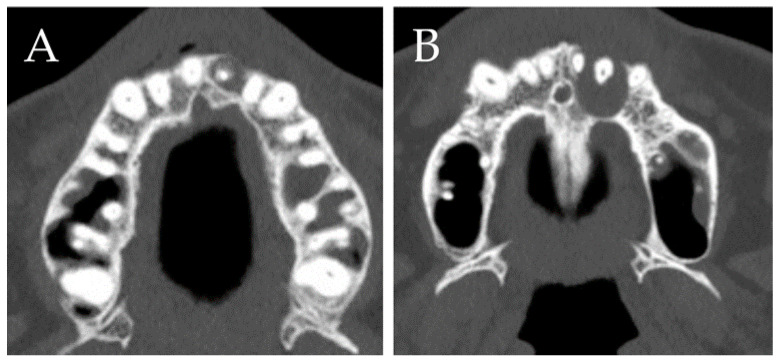
Representative perilesional sclerotic images in axial view of CT: (**A**) positive perilesional sclerotic sign; (**B**) negative perilesional sclerotic sign.

Predictor variables for the study comprised sets of exposures considered convincingly related to endodontic surgery success rates. These factors were divided into demography, health anatomical, pathological, operative variables, and dental status (restoration and post). While demographic variables included the gender and age of the patients, health status measures included a history of diabetes, chronic hepatitis, malignant tumor, and intake of corticosteroids. Anatomical variables included tooth position data (anterior, premolar, and molar, maxilla/mandible, left/right). Pathological variables included the lesion size, expansion of the jaw, cortical bone defect, and presence of a perilesional sclerotic sign (Figure 2), including through-and-through bone defects. Operative variables included the selection of bone lid surgery [10,11]. Dental variables included restoration type (bridge/single/splinted crown) and the presence or absence of post.

### 2.6. Outcome Variables

Patients were typically followed up at 2 weeks and 3, 6, and 12 months. At 6 and 12 months, panoramic radiographs and CT were conducted. A subsequent evaluation of results was performed if the tooth evaluation showed subjective discomfort, swelling, sinus tract, or loss of function (classified as a clinical failure). Alternatively, healing patterns on radiographs and CT images were classified on the basis of the report by Kang et al. [12]. If the tooth showed complete or limited healing without clinical failure, it was grouped under ‘healing.’ However, teeth were grouped under “failure” if they showed clinical failure despite the radiographic and CT imaging evaluation results.

### 2.7. Statistical Analysis

Data were recorded in an electronic database throughout the study using Microsoft Excel (Microsoft Inc., Redmond, WA, USA). For statistical analysis, the digital database used was JMP version 14.2.0 for Macintosh (SAS Institute Inc., Cary, NC, USA). First, the mean and standard deviations (SD) were used for continuous variables. Parametric and nonparametric tests (*t*-test and Pearson chi-square) compared the success and failure groups. Any association in bivariate analyses with *p* < 0.05 was included in a multiple logistic regression analysis, which was subsequently used to provide adjusted odds ratios (ORs) to control the simultaneous effects of multiple covariates. Significant differences were set at a level of *p* < 0.05.

The correlation coefficient is an index that measures the strength of a linear relationship between two data points or a random variable. In the range of 0 to 1, the success rate of endodontic surgery increased with increasing index values. Subsequently, correlation charts were plotted according to variable data based on the number of successful surgeries. Then, the algorithm for calculating statistical properties was programmed in Python (version 3.7.12) using NumPy (version 1.19.5) and the pandas library (version 1.3.5) for statistical analysis.

## 3. Results

Of the 479 patients in the cohort, 242 were used to assess outcomes and prognostic factors. The 1 year success rate of endoscopic endodontic treatment was 95.3% (345/362). The sample breakdown for this study is summarized in Table 1.

### 3.1. Distribution of Predictive Variables Based on the Results Obtained after Endodontic Surgeries

Table 2 shows the distribution of predictive variables, following the results obtained after endoscopic endodontic surgeries. Variables statistically related to good healing after endodontic surgery (*p* < 0.05), including age (*p* = 0.007), perilesional sclerotic sign (*p* = 0.022), and the presence of posts (*p* < 0.001), are shown. There were no statistically significant differences in any of the health status or operative variables. In examining the correlation coefficients accounting for the prognosis of endodontic surgery, the top 3 factors were the presence or absence of a post, perilesional sclerotic signs, and age.

### 3.2. Correlation Coefficients between Endodontic Surgical Success and Failure Variables

**Figure 3:**
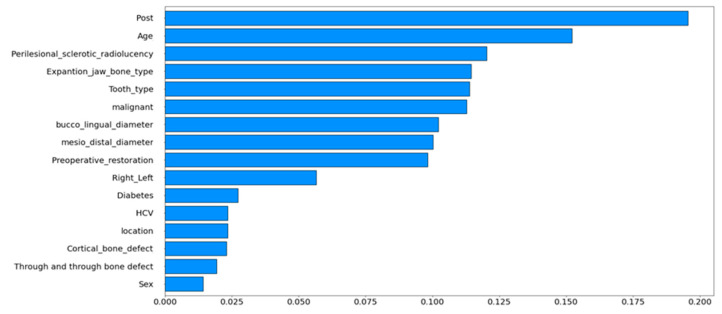
Diagram showing the correlation coefficients between endodontic surgical success and failure variables. The influence of the correlation coefficients is shown.

Figure 3 shows the correlation coefficients for the clinical results of endodontic surgery in a bar graph. Similarly, the strongest correlations for the clinical results for endoscopic endodontic surgery were post, perilesional sclerotic signs, and age.

### 3.3. Multivariate Logistic Regression Model Results of Endodontic Surgery

Subsequently, we adopted a multivariate logistic regression model containing candidate variables identified as the strongest correlations from the clinical endoscopic endodontic surgery results using a bivariate analysis and biologically important variables. Therefore, age (OR = 3.107, *p* = 0.145), perilesional sclerotic signs (OR = 7.348, *p* = 0.066), and the presence or absence of posts (OR = 9.592, *p* < 0.001) were used. The results showed that the presence of perilesional sclerotic signs and posts were associated with an increased risk of failure after endodontic surgery (Table 3).

## 4. Discussion

This retrospective study investigated the success rate and risk factors accounting for successful endoscopy-assisted-endodontic surgery. The success rate of endodontic surgery was 95.3%, showing the highly predictable nature of the treatment. Additionally, of the clinical variables, the presence of a post was the strongest risk factor affecting endodontic surgery in our study.

After introducing the principle of microsurgery for endodontic surgery, continuous research for the further enhanced visualization of the surgical field has been observed [13]. Furthermore, the success rates for endodontic surgery using an endoscope in previous studies were 88.9–94.9% [3,4,5,14]; in our study the success rate was 95.3%, which was about the same. Additionally, it was difficult to compare the success rates of endodontic surgeries performed at different institutions. This difficulty was due to slight differences in surgical procedures, the surgeon’s technique, and the surgical devices [15]. Additionally, it was fascinating to observe high predictability. Endoscopic visualization has also become the standard of care in modern medicine for diagnosis and surgical treatment. Thus, introducing an endoscope gives good results in the maxillofacial region [16,17,18]. The major advantages of endoscopic surgeries are the possibility of securing a reliable surgical field of view through the magnifying effect and the possibility of sharing the surgical field of view. Sharing the professional field of view contributes to shortening the learning curve for surgery. Additionally, the benefits of using endoscopic surgery in endodontic surgeries avoid multiple adjustments typical of traditional overhead dental surgery lights compared to loupes [3]. Therefore, compared with a microscope, an endoscope is easier to use and can be used regardless of the angle of the patient’s face. The range of use is also extensive. On the other hand, there is a limitation of endoscopic assisted-endodontic surgery compared to microscopic endodontic surgery. The endoscope cannot be operated by the surgeon during surgery. The first assistant operates the endoscope. Therefore, continuous team education and growth are required for smooth and accurate surgery.

In this study, MTA cement was used as a root-end filling material for endodontic surgery. Previous studies has reported the use of MTA with a higher healing rate compared to other root end filling materials [19,20]. MTA cement, which is a bioceramic material, is a ceramic material that has excellent biocompatibility and excellent sealing properties and is not affected by contamination with blood or periradicular fluid [21]. It also increases cell proliferation and promote mineralization [22]. This biomaterial will have a significant impact on the success rate of endodontic surgery.

In clinical studies to date, the maxilla has shown a higher healing rate in the anterior teeth than the mandible, especially since the mandibular molars have low healing rates [19,23]. This difference is due to the easier surgical access to the maxilla and anterior teeth than the mandibular molars [20]. Interestingly, however, although our study observed no statistically significant difference between the maxilla and mandible, the correlation between the anterior and molars was low. These results suggest that the endoscope was effective against anatomical restrictions. 

It has been reported that defects in osteosclerotic signs are risk factors accounting for endodontic surgery. Osteosclerotic signs show an inflammatory condition [24]. The oral cavity is constantly exposed to bacteria compared to the outside of the oral cavity, and the jawbone is susceptible to bacterial infection [25]. Acute clinical symptoms, such as inflammation-induced pain and swelling, have also been associated with the disappearance of perilesional sclerotic radiolucency around the lesion [26]. It has also been speculated that the disappearance of osteosclerosis is associated with a stage of acute infection, impairing surgical wound healing. Preoperative pain is also involved in the prognosis of endodontic surgery [19], consistent with previous reports that the disappearance of osteosclerosis in a previous study was a risk factor for endodontic surgeries.

The study revealed that the presence of posts was statistically significant and highly correlated with the prognosis of endodontic surgery. Past studies have reported that the presence or absence of posts was not statistically or significantly involved in healing. However, although no statistically significant differences exist in many reports [19], the presence of posts reduces success rates [3,27,28,29]. Similarly, this study suggested that the presence of posts is a risk factor for failure, affecting surgical prognosis. Current recommendations for endodontic surgery include the formation of a cavity 3 mm lengthwise from the apex and 3 mm deep. A post that limits the therapeutic area sufficiently for endodontic surgery can impair the preparation and occlusion of the root cavity. However, the sample size in this study was small, indicating a bias for patients who have undergone surgery. Therefore, further studies with an increased number of subjects will be necessary.

This study used the correlation coefficient as a statistical evaluation tool to identify prognostic factors accounting for endodontic surgery. Although the number of relationships is a measure of the linear relationships between random variables [30], it does not explain the causal relationships between random variables. Furthermore, even though the correlation coefficient is an ordinal scale, not a ratio scale, it is impossible to simply compare the degree of strength. Therefore, it is necessary to identify the most substantial risk factor from the odds ratio through multivariate analysis. In this study, the rank of the correlation coefficient was similar to the odds ratio rank. The advantage of calculating the correlation coefficient is that the correlation can be quickly and visually evaluated. Additionally, this paper is the first to identify risk factors for endodontic surgery using a correlation coefficient, and its novelty is proposed to be meaningful.

Four limitations were encountered during this study. The first limitation was that the number of samples was too small, as mentioned above. However, the success rate of endodontic surgery was high. Therefore, it is necessary to increase the number of cases in future studies. The second limitation was the short follow-up period. In this study, the evaluation was based on a follow-up period of 1 year after surgery. Future research will require reporting on the results of medium-term follow-up. The third limitation was the type of predictive variable. Oral hygiene is an important risk factor associated with oral and maxillofacial surgery [31]. The oral mucosa is also affected by the general condition [32]. Unfortunately, this study did not evaluate oral hygiene. Further research is needed as a future research topic. The fourth limitation was that our research was a single facility study. Hence, the possibility that the case selection will be strongly biased exists. To reduce the bias, it is necessary to change the research design to a multicenter research type

## 5. Conclusions

This retrospective study revealed the 1 year success rate and risk factors accounting for successful endoscopic endodontic surgeries. The success rate of endodontic surgery was 95.3%, proposing it as a highly predictable treatment. Among the clinical variables, the presence of posts was the strongest risk factor accounting for endodontic surgery successes. Additionally, we identified risk factors using the correlation coefficient and determined the importance of risk factors based on the odds ratio using multivariate analysis.

## Figures and Tables

**Table 1:** Number of understudied patients and their endoscopic endodontic surgery success rates.

Patients				
Gender	Male	Female	Total	
	90	152	242	
%	37.2	62.8		
Age (years)	Mean	SD	Range	Median
	48.3	15.0	12- 82	49.0
Outcome	n	%		
Success	345	95.3		
Failure	17	4.7		

**Table 2:** Compared distribution of predictive variables based on the results obtained after endodontic surgeries.

	Failure	Success	*p*-Value
Outcome (%)	17 (4.7)	345 (95.3)	
**Demographic variables**			
Age			
>60	9	77	0.007
≤60	8	268	
Gender			0.788
Male	6	133	
Female	11	212	
**Health status variables**			
Diabetes	1	12	0.603
Chronic hepatitis	0	4	0.655
Malignant tumor	2	9	0.089
**Anatomic variables**			
Tooth position			0.095
Anterior	9	259	
Premolar	5	62	
Molar	3	24	
Jaw			0.774
Maxilla	12	260	
Mandible	5	85	
Lt/Rt			0.325
Lt	7	188	
Rt	10	157	
**Pathological variables**			
Mesio-distal			0.096
Large	6	59	
Small	11	286	
Bucco-lingual			0.076
Large	6	204	
Small	11	141	
Expansion of the jaw			0.029
Presence	0	76	
Absence	17	269	
Cortical bone defect			0.797
Presence	10	221	
Absence	7	124	
Perilesional sclerotic signs			0.022
Presence	1	111	
Absence	16	234	
Through-and-through bone defect			0.521
Presence	1	14	
Absence	16	331	
**Operative variables**			
Bone lid surgery			0.583
Presence	0	17	
Absence	6	338	
**Dental variables**			
Restoration			0.062
bridge	3	21	
Single/splinted crown	14	324	
Post			<0.001
Presence	15	146	
Absence	2	199	

**Table 3:** Multivariate logistic regression model results of endodontic surgery.

	Standard Error	OR	Lower 95%	Upper 95%	*p* Value
Post	0.383	9.592	2.135	43.092	<0.001
Perilesional sclerotic signs	0.523	7.348	0.880	53.386	0.066
Age	0.263	3.107	1.118	8.635	0.030

## Data Availability

The datasets used and/or analyzed during the current study are available from the corresponding author upon reasonable request.

## References

[1] Kim E., Kim Y. (2019). Endodontic microsurgery: Outcomes and prognostic factors. Curr. Oral Health Rep..

[2] Tawil P.Z., Saraiya V.M., Galicia J.C., Duggan D.J. (2015). Periapical microsurgery: The effect of root dentinal defects on short- and long-term outcome. J. Endod..

[3] Taschieri S., del Fabbro M., Testori T., Francetti L., Weinstein R. (2006). Endodontic surgery using 2 different magnification devices: Preliminary results of a randomized controlled study. J. Oral Maxillofac. Surg..

[4] Taschieri S., del Fabbro M., Testori T., Weinstein R. (2008). Microscope versus endoscope in root-end management: A randomized controlled study. Int. J. Oral Maxillofac. Surg..

[5] Taschieri S., del Fabbro M. (2009). Endoscopic endodontic microsurgery: 2-year evaluation of healing and functionality. Braz. Oral Res..

[6] Pallarés-Serrano A., Glera-Suarez P., Tarazona-Alvarez B., Peñarrocha-Diago M., Peñarrocha-Diago M., Peñarrocha-Oltra D. (2021). Prognostic Factors after Endodontic Microsurgery: A Retrospective Study of 111 Cases with 5 to 9 Years of Follow-up. J. Endod..

[7] Blahuta R., Stanko P. (2012). The use of optical magnifying devices in periradicular microsurgery. Bratislava Med. J..

[8] Baldassari-Cruz L.A., Lilly J.P., Rivera E.M. (2002). The influence of dental operating microscope in locating the mesiolingual canal orifice. Oral Surg. Oral Med. Oral Pathol. Oral Radiol. Endod..

[9] Filho T.C., Sá La Cerda R., Filho E.D.G., de Deus G.A., Magalhães K.M. (2006). The influence of the Surgical Operating Microscope in locating the mesiolingual canal orifice: A laboratory analysis. Braz. Oral Res..

[10] Sukegawa S., Yamamoto N., Matsuyama T., Takabatake K., Kawai H., Nagatsuka H., Furuki Y. (2021). Factors of Successful Treatment Using the Bone Lid Technique in Maxillofacial Surgery: A Pilot Study. J. Hard Tissue Biol..

[11] Sukegawa S., Kanno T., Kawai H., Shibata A., Matsumoto K., Sukegawa-Takahashi Y., Sakaida K., Nagatsuka H., Furuki Y. (2016). Surgical Treatment and Dental Implant Rehabilitation after the Resection of an Osseous Dysplasia. J. Hard Tissue Biol..

[12] Kang S., Ha S.W., Kim U., Kim S., Kim E. (2020). A One-Year Radiographic Healing Assessment after Endodontic Microsurgery Using Cone-Beam Computed Tomographic Scans. J. Clin. Med..

[13] Del Fabbro M., Taschieri S. (2010). Endodontic therapy using magnification devices: A systematic review. J. Dent..

[14] Taschieri S., del Fabbro M., Testori T., Weinstein R. (2007). Endoscopic periradicular surgery: A prospective clinical study. Br. J. Oral Maxillofac. Surg..

[15] Zuolo M.L., Ferreira M.O.F., Gutmann J.L. (2000). Prognosis in periradicular surgery: A clinical prospective study. Int. Endod. J..

[16] Kishimoto T., Sukegawa S., Ono S., Nakamura S., Ando M., Yoshino T., Furuki Y. (2021). Endoscope-assisted enucleation of mandibular dentigerous cysts. J. Oral Maxillofac. Surgery, Med. Pathol..

[17] Kishimoto T., Sukegawa S., Katase N., Kanno T., Sukegawa-Takahashi Y., Masui M., Sato A., Furuki Y. (2019). Endoscope-assisted resection of intramuscular cavernous hemangioma within the temporal muscle. J. Craniofac. Surg..

[18] Hayashi H., Abe A., Ota M., Momokita M., Ishihama T., Furuta H., Taniguchi T., Takeuchi K. (2021). Endoscopic removal of accidental aspirated and ingested dental foreign bodies: A cross-sectional study. Medicine.

[19] Von Arx T., Peñarrocha M., Jensen S. (2010). Prognostic factors in apical surgery with root-end filling: A meta-analysis. J. Endod..

[20] Bliggenstorfer S., Chappuis V., von Arx T. (2021). Outcome of Periapical Surgery in Molars: A Retrospective Analysis of 424 Teeth. J. Endod..

[21] Kim S., Song M., Shin S.J., Kim E. (2016). A randomized controlled study of mineral trioxide aggregate and super ethoxybenzoic acid as root-end filling materials in endodontic microsurgery: Long-term outcomes. J. Endod..

[22] Bonson S., Jeansonne B.G., Lallier T.E. (2004). Root-end filling materials alter fibroblast differentiation. J. Dent. Res..

[23] Song M., Jung I.Y., Lee S.J., Lee C.Y., Kim E. (2011). Prognostic factors for clinical outcomes in endodontic microsurgery: A retrospective study. J. Endod..

[24] Dunfee B.L., Sakai O., Pistey R., Gohel A. (2006). Radiologic and pathologic characteristics of benign and malignant lesions of the mandible. Radiographics.

[25] Vinci R., Teté G., Lucchetti F.R., Capparé P., Gherlone E.F. (2019). Implant survival rate in calvarial bone grafts: A retrospective clinical study with 10 year follow-up. Clin. Implant Dent. Relat. Res..

[26] Sukegawa S., Matsuzaki H., Katase N., Kawai H., Kanno T., Asaumi J.I., Furuki Y. (2020). Morphological characteristics of radicular cysts using computed tomography. Odontology.

[27] Maddalone M., Gagliani M. (2003). Periapical endodontic surgery: A 3-year follow-up study. Int. Endod. J..

[28] Taschieri S., del Fabbro M., Testori T., Francetti L., Weinstein R. (2005). Endodontic surgery with ultrasonic retrotips: One-year follow-up. Oral Surg. Oral Med. Oral Pathol. Oral Radiol. Endod..

[29] Rahbaran S., Gilthorpe M.S., Harrison S.D., Gulabivala K. (2001). Comparison of clinical outcome of periapical surgery in endodontic and oral surgery units of a teaching dental hospital: A retrospective study. Oral Surg. Oral Med. Oral Pathol. Oral Radiol. Endod..

[30] Mari D.D., Kotz S. (2001). Correlation and Dependence.

[31] Tecco S., Grusovin M.G., Sciara S., Bova F., Pantaleo G., Capparé P. (2018). The association between three attitude-related indexes of oral hygiene and secondary implant failures: A retrospective longitudinal study. Int. J. Dent. Hyg..

[32] Rosso M., Blasi G., Gherlone E., Rosso R. (1997). Effect of granulocyte-macrophage colony-stimulating factor on prevention of mucositis in head and neck cancer patients treated with chemo-radiotherapy. J. Chemother..

